# Prospective Longitudinal Evaluation of the Peripheral Choroidal Thickness in High-Risk Infants with Retinopathy of Prematurity: A Pilot Study.

**DOI:** 10.1155/2020/4384328

**Published:** 2020-05-23

**Authors:** Mohamed A. Hussein, Evelyn Paysse

**Affiliations:** Department of Ophthalmology, Texas Children's Hospital and Baylor College of Medicine, Houston, TX, USA

## Abstract

**Objective:**

The purpose of this study was to prospectively follow up the peripheral choroidal development and thickness for retinopathy of prematurity (ROP) in high-risk premature infants using optic coherence tomography.

**Materials and Methods:**

All infants included in the analysis had an optical coherence tomography (OCT) evaluation, serially over 6 weeks, starting at the first ROP screening exam and at each follow-up screening exam. We included infants born at or earlier than 25 weeks of gestation, weighing less than 700 grams, and those who developed interventricular hemorrhage. We evaluated the choroidal thickness and development, centrally and at the extreme nasal and temporal periphery, for each eye with each ROP screening exam. Changes in the choroidal thickness and the choroidal thickness to retinal thickness ratio (C/R ratio) were evaluated over time not only for each individual infant, but between the infants as well.

**Results:**

Six infants met our inclusion criteria. Infants with severe ROP had a mean choroidal thickness that was significantly thinner in the extreme temporal periphery than infants that did not develop significant ROP (*P*=0.02). The mean value of the C/R ratio was smaller in infants with severe ROP compared to those that did not develop any significant ROP at any of the evaluated locations (*P* < 0.001).

**Conclusion:**

The peripheral choroid appears to be significantly thinner relative to the retinal thickness in infants with severe ROP.

## 1. Introduction

The choroid provides oxygen and nutrition to the majority of the retina in term infants [1]. The peripheral choroid is intuitively responsible for supplying the entirety of the peripheral retina with oxygen and nutrients until retinal vascular development occurs in premature infants [2, 3]. We previously proposed a novel hypothesis explaining the development of retinopathy of prematurity (ROP) based on the lack of autonomic innervation in premature infants [2, 3]. The peripheral choroid, a major source of the blood supply to the outer retina, may play an important role in the development of ROP [2, 3]. Premature infants are known to suffer a major deficit in autonomic nervous system (ANS) activity: the more premature the neonate, the more likely that the ANS will be dysfunctional [4]. Unlike the retinal vasculature, which is largely autoregulated, choroidal perfusion is largely controlled via the ANS [5]. Defective ANS activity in premature infants is thought to lead to dysregulation of choroidal perfusion, which may adversely affect the blood supply resulting in peripheral retina hypoxia and ROP development [2, 3]. We have found that the ANS, particularly the parasympathetic arm, is severely deficient in infants that develop severe ROP requiring treatment compared to similar high-risk infants who do not develop significant ROP [3]. Since choroidal perfusion is known to be autonomically innervated, the abnormality of ANS activity in premature infants is expected to affect the thickness and the development of the choroid. To further elaborate on this theory, we elected to measure the peripheral choroidal thickness and to study the choroidal development in relation to retinal development. We then followed these developmental changes over time using handheld spectral domain optical coherence tomography (SD-OCT) in a series of very high-risk premature infants. Multiple studies have evaluated the central choroidal thickness in high-risk premature infants using OCT [6–10]. This, we believe, is the first study evaluating the peripheral choroid in very high-risk premature infants using SD-OCT. The purpose of this study was to evaluate the peripheral choroidal thickness in relation to the overlying retina in high-risk premature infants who developed ROP and needed treatment. We then compared the results to infants with similar risk factors who did not develop significant ROP.

## 2. Materials and Methods

This study was approved by the Institutional Review Board of Baylor College of Medicine. The Ethical Committee approval number is H-36754. Parents or legal guardians provided informed consent for each subject to participate.

We prospectively followed up the development and thickness of the peripheral choroid in infants at high risk for ROP using handheld SD-OCT. We included premature infants who met the criteria for having a very high risk of developing severe ROP. To be included in the study, premature infants had to be born at or earlier than 25 weeks of gestation, weighing less than 700 grams, and must have developed interventricular hemorrhage. Preterm infants who met the inclusion criteria but who had severe systemic conditions and eye disease other than ROP were excluded from the study. Also excluded from the study were infants whose images obtained did not go beyond the anatomical macula or if the images did not have a clear choroid-scleral junction (CSJ) at the periphery to allow for proper measurement of the choroidal thickness. Infants who did not have serial imaging for at least 6 weeks were excluded from the analysis as well. Preterm infants underwent standard ROP screening to obtain data such as zone, stage, and vascular status as per the routine ROP screening protocol. SD-OCT imaging was performed before or after ophthalmic examination on supine infants in the NICU incubator without sedation and without an eyelid speculum using a portable handheld SD-OCT (Leica Envisu-C Class 2300 SD-OCT, Bioptigen, Inc.). When necessary, proparacaine would be administered to the infant and a depressor was used to rotate the eye to scan the far periphery.

At least 3 scans were taken for each eye during each exam session with all infants being imaged using SD-OCT, serially and longitudinally, starting at the initial ROP screening exam and at each follow-up ROP screening exam. Follow-up imaging continued serially until either the infant developed ROP severe enough to require treatment or until they were discharged from the NICU. All SD-OCT images were converted into high-quality digital images; however, only the highest quality peripheral choroidal frame from each screening exam was chosen for qualitative and quantitative analysis. OCT scan images were considered for analysis only if they had a clear CSJ to allow for proper measurement of the choroidal thickness, which was measured centrally (at least 5-disc diameters (DD) from the optic nerve superiorly or inferiorly). As it was difficult to image the choroid and retinal periphery at the extreme periphery with good quality both superiorly and inferiorly, we elected to use the highest quality image with the most distinct CSJ for quantitative and qualitative analysis. For simplicity, we referred to the latter images as central. The choroidal thickness at the nasal and temporal periphery was also measured for each eye (at least 5 DD temporal to the temporal margin of the optic nerve; 5 DD nasal to the nasal margin of the optic nerve; and as far peripherally as the image allowed for clear visualization of the CSJ). Although we measured the choroidal thickness at the various locations, there was no universal reference point for the choroidal periphery to allow for proper comparison of the choroidal thickness in the serial scans of the same patient or between the images of different patients. Accordingly, we elected to compare the choroid thickness to retina thickness ratio (C/R ratio) in each infant and between the individual infants over time. The C/R ratio was correlated with ROP exam findings.

According to the ROP screening results, the infants were divided into the following groups based on the severity of ROP at any of the serial examinations: (group 1) type I ROP requiring treatment; (group 2) severe ROP of stage 2 or worse but not requiring treatment; and (group 3) mild ROP of </ = stage 1 in either eye at any screening exam. The most peripheral choroidal location in each session and for each eye that had clear CSJ was used for analysis. The mean of the choroidal thickness and the mean of the C/R ratio was calculated for both eyes together and for all serial images of each patient in each of the 3 locations (temporally, nasally, and centrally). We compared the mean of the choroid thickness and the mean (C/R) ratio between the 3 individual groups at the 3 designated locations.

## 3. Statistical Analysis

We performed standard statistical testing of the test results, including calculation of descriptive statistics, mean and standard deviation.

Analysis of variance (ANOVA) was used to compare the mean of the choroid thickness and the mean (C/R) ratio between the 3 individual groups at the 3 designated locations. A *P* value of less than 0.05 was considered to be statistically significant.

The ANOVA test is used to determine whether there are any statistical significant differences among three or more independent groups.

## 4. Results

**Figure 1:**
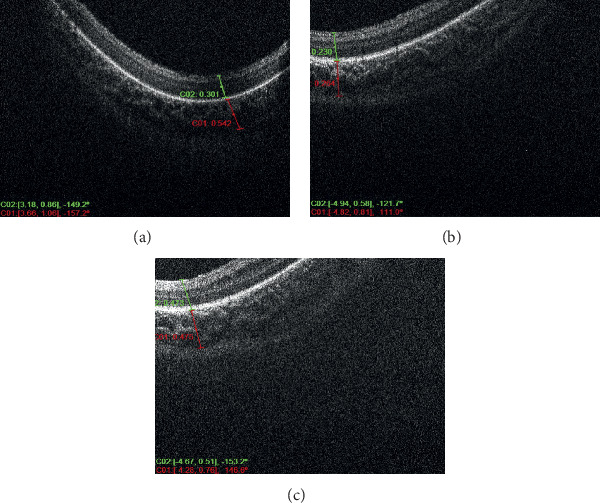
Serial OCT photos of the right eye of a high-risk premature infant, who had very mild ROP, showing a choroid that is thicker than the overlying retina.

**Figure 2:**
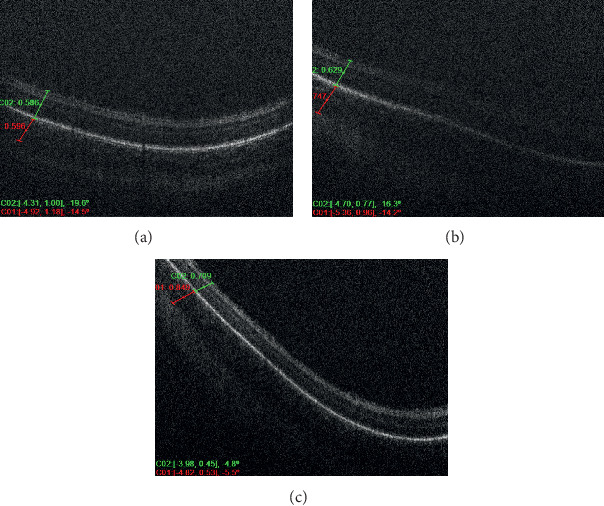
Serial OCT of the right eye of a high-risk premature infant, who had mild ROP disease, demonstrating thicker choroid in relation to the overlying retina.

**Figure 3:**
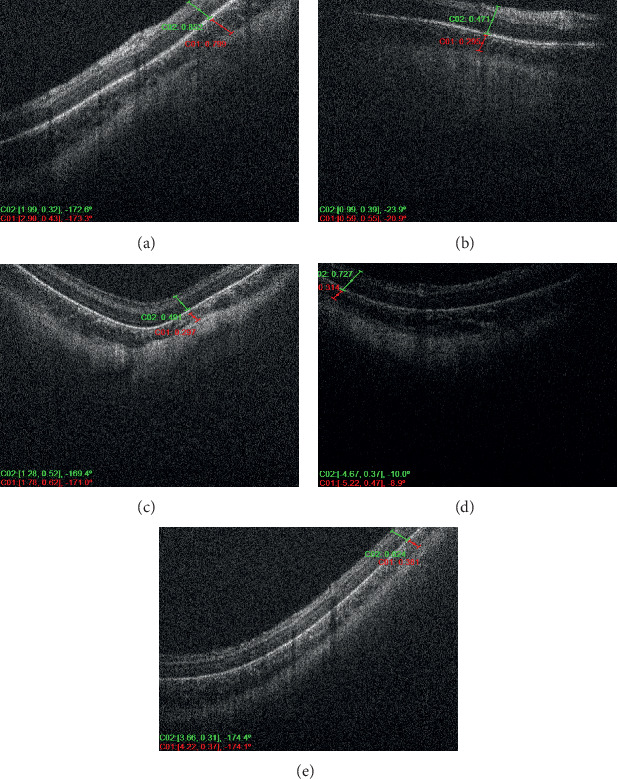
Serial OCT photos of the right eye of a high-risk premature infant, who developed severe ROP and needed treatment, showing choroid that is thinner than the overlying retina.

**Figure 4:**
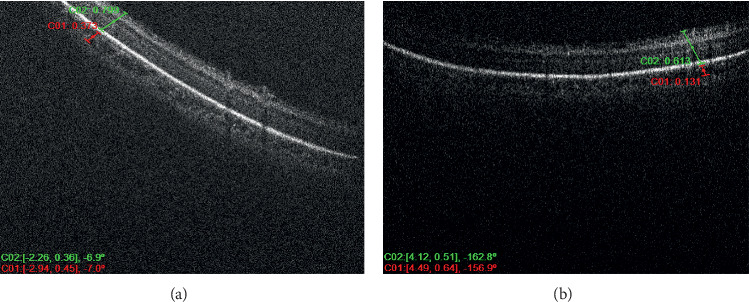
OCT of the right eye (to the right) and left eye (to the left) of a high-risk premature infant that ended up needing treatment, showing a thinner choroid in relation to the overlying retina in both eyes.

Six infants met the inclusion criteria of the study and were divided among the groups, where each of the three groups consisted of two infants a piece. Each included patient of the study had at least 3 serial OCT scan sessions over a period of at least 6 weeks with at least 1-week duration in between the serial scans. Each patient had an average of 4 imaging sessions for both eyes; however, the discrepancy in the number of sessions between the different patients was related to the fact that some of the OCT imaging sessions were omitted or not included because of the inability to determine with certainty the location of the CSJ. Figures 1 and 2 demonstrate OCT images of premature infants who had very mild ROP, showing the peripheral choroid being thicker than the overlying retina (high C/R ratio). Figures 3 and 4 demonstrate OCT images for a high-risk premature infant who developed severe ROP and needed treatment, showing a peripheral choroid that is thinner than the overlying retina (low C/R ratio). The mean choroidal thickness and choroid to retina thickness ratio (C/R ratio) at the 3 locations for all 3 groups are presented in Table 1. The mean choroidal thickness centrally (superiorly or inferiorly) was 308 ± 144, 394 ± 197, and 438 ± 217 in groups 1, 2, and 3, respectively. The mean choroidal thickness centrally was larger in the mild ROP group compared to the severe ROP and treated groups. This, however, did not reach statistical significance (*P*=0.3). The C/R ratio, centrally, was 0.6 ± 0.1, 0.6 ± 0.1, and 1.4 ± 0.2 in groups 1, 2, and 3, respectively. The C/R ratio was significantly larger in the mild ROP group compared to the severe ROP and treated groups (*P* < 0.0001). The mean choroidal thickness temporally was 361 ± 138, 535 ± 141, and 511 ± 76 in groups 1, 2, and 3, respectively. The mean choroidal thickness temporally was statistically larger in the mild ROP group and severe nontreated group compared to the treated ROP infants (*p*=0.02). The C/R ratio, temporally, was 0.6 ± 0.2, 0.7 ± 0.1, and 1.3 ± 0.2 in groups 1, 2, and 3, respectively. The C/R ratio temporally was significantly larger in the mild ROP group compared to the severe ROP and treated groups (*P* < 0.0001). The mean choroidal thickness nasally was 426 ± 128, 540 ± 271, and 552 ± 94 in groups 1, 2, and 3, respectively. The mean choroidal thickness was larger in the mild ROP group compared to the severe ROP and treated groups. This, however, did not reach statistical significance (*P*=0.2). The C/R ratio, centrally, was 0.6 ± 0.1, 0.8 ± 0.1, and 1.4 ± 0.3 in groups 1, 2, and 3, respectively. The C/R ratio nasally was significantly larger in the mild ROP group compared to the severe ROP and treated groups (*P* < 0.0001).

## 5. Discussion

We have reported that an immature and deficient ANS, particularly the parasympathetic arm of the ANS system, is associated with the development of severe ROP and may play a role in its development [2, 3]. Choroidal perfusion is controlled by the ANS, particularly the parasympathetic activity, while the retinal perfusion is largely autoregulated [1]. As such, the deficiency of ANS activity in the eyes of premature infants should mainly affect choroidal perfusion [3]. The lack of properly regulated choroidal perfusion, particularly the periphery, could be an important factor in producing hypoxia in the peripheral retina, in turn leading to or contributing to the development of ROP [2, 3].

Multiple studies have evaluated the choroidal thickness in premature infants using OCT [6–10]. These studies demonstrated a thinner choroid in premature infants that develop severe ROP; however, only the central choroidal thickness was evaluated and most of these studies were conducted in older age groups than our cohort [9, 10]. Since the central retina is typically vascularized in most premature infants, this study concentrated more on evaluating the peripheral choroid of which, we believe, may be more involved in the development of ROP [2, 3]. According to our research, our current study is the first to evaluate the peripheral choroid in a group of high-risk premature infants using SD-OCT. The purpose of this pilot study was to determine if the peripheral choroid had any anatomical difference in high-risk premature infants who developed ROP severe enough to need treatment compared to preterm infants with similar very high-risk criteria who did not develop severe ROP. Imaging and evaluation of the central choroid is technically easier given the central location, and its clearer anatomical landmarks as compared to the peripheral choroid. Because of the lack of fixed peripheral anatomical landmarks for both the retina and the choroid, the research showed that measuring the ratio of the thickness of the choroid to the thickness of the overlying retina (C/R ratio) will be a more reliable index for evaluation rather than measuring the peripheral choroidal thickness alone.

The study demonstrated that the peripheral choroid appears to be thinner on serial OCT scans in high-risk premature infants who developed severe ROP requiring treatment compared to the ones that had similar risk factors but did not develop significant ROP. Similarly, the C/R ratio was significantly smaller in high-risk premature infants who developed severe ROP requiring treatment compared to the group with similar risk factors that did not develop significant ROP. Although previous studies have evaluated the thickness of the central choroid to try to establish normative values [8], no study has attempted to determine normative values for the thickness of the peripheral choroid. Because the peripheral retinal, however, lacks any consistent landmarks visible on OCT which would allow reliable repeatable evaluations of the same peripheral choroidal and retinal thickness, we believe that the C/R ratio is a more reliable index.

One limitation of this study is the small number of infants in this cohort. This was a pilot study to determine if our study hypothesis was sound and if the question merits further investigation. We believe that although the number may have been small, the infants studied had enough serial OCT scans over a reasonable period of time to show definite differences and demonstrated distinctively different anatomical ROP outcomes, sufficiently so to allow highly suggestive statistical results. We believe that further studies exploring the role of the periphery of the choroid in the development and severity of ROP are warranted. Another limitation of the study is that the choroidal thickness as measured by SD-OCT may not be reflective of the actual choroidal perfusion. Future studies, using angiographic evaluation of the periphery of the choroid, including (OCTA), may be better in evaluating the choroidal perfusion.

In conclusion, the peripheral choroid appears to be significantly thinner relative to the retinal thickness in infants with severe ROP than similarly premature infants with mild ROP.

## Figures and Tables

**Table 1:** The mean ± standard deviation choroidal thickness and choroid to retina thickness ratio (C/R ratio) at the 3 locations for all 3 groups.

ROP severity	Central thickness (*μ*)	Central C/R	Temporal thickness (*μ*)	Temporal C/R	Nasal thickness (*μ*)	Nasal C/R
Mild	438 ± 217	1.4 ± 0.2	511 ± 76	1.3 ± 0.2	552 ± 94	1.4 ± 0.3
Severe ROP	394 ± 197	0.6 ± 0.1	535 ± 141	0.7 ± 0.1	540 ± 271	0.8 ± 0.1
ROP requiring treatment	308 ± 144	0.6 ± 0.1	361 ± 138	0.6 ± 0.2	426 ± 128	0.6 ± 0.1
*P* value	0.3	<0.0001	0.02	<0.0001	0.2	<0.0001

## Data Availability

The clinical data used to support the findings of this study are included within the article.
